# The incidence of acute oxaliplatin-induced neuropathy and its impact on treatment in the first cycle: a systematic review

**DOI:** 10.1186/s12885-018-4185-0

**Published:** 2018-04-12

**Authors:** Endale Gebreegziabher Gebremedhn, Peter John Shortland, David Anthony Mahns

**Affiliations:** 1 School of Medicine, Western Sydney University, Locked Bag 1797, Penrith NSW, Sydney, 2751 Australia; 2 School of Science and Health, Western Sydney University, Locked Bag 1797, Penrith NSW, Sydney, 2571 Australia

**Keywords:** Colorectal cancer, Oxaliplatin, Acute neuropathy, Chronic neuropathy

## Abstract

**Background:**

Although acute oxaliplatin-induced neuropathy (OXIPN) is frequently regarded to be transient, recent studies have reported prolongation of infusion times, dose reduction and treatment cessation following the first dose of oxaliplatin in quarter of patients. Acute OXIPN is also a well-established risk factor for chronic neuropathy. However, there is underreporting of these parameters during the acute phase (≤ 14 days). This paper systematically reviews the incidence of acute OXIPN and its impact on treatment in the first cycle.

**Methods:**

A systematic literature search was performed using PubMed and Medline. Published original articles were included if they described details about prevalence of oxaliplatin-induced acute neuropathy.

**Results:**

Fourteen studies, comprised of 6211 patients were evaluated. The majority of patients were treated with oxaliplatin in combination with leucovorin and fluorouracil (FOLFOX). Most studies used the National Cancer Institute Common Toxicity Criteria to assess acute neuropathy. Acute neuropathy (Grades 1–4) was the most common event with prevalence ranging from 4–98%, followed by haematological (1.4–81%) and gastrointestinal (1.2–67%) toxicities, respectively. Drug regimens, starting dose of oxaliplatin and neuropathy assessment tools varied across studies. In addition, moderate to severe toxicities were common in patients that received a large dose of oxaliplatin (> 85 mg/m^2^) and/ or combined drugs. The majority of studies did not report the factors affecting acute neuropathy namely the range (minimal) doses required to evoke acute neuropathy, patient and clinical risk factors. In addition, there was no systematic reporting of the number of patients subjected to prolonged infusion, dose reduction, treatment delay and treatment cessation during the acute phase.

**Conclusion:**

Despite the heterogeneity of studies regarding oxaliplatin starting dose, drug regimen, neuropathy assessment tools and study design, a large number of patients developed acute neuropathy. To develop a better preventive and therapeutic guideline for acute/chronic neuropathy, a prospective study should be conducted in a large cohort of patients in relation to drug regimen, starting/ranges (minimal) of doses producing acute neuropathy, treatment compliance, patient and clinical risk factors using a standardised neuropathy assessment tool.

## Background

Globally, colorectal cancer (CRC) is a major public health problem [1, 2]. CRC is the third most common cancer in men and the second in women worldwide and the incidence is rising in many countries [3]. Surgery is the main curative therapy for stage II and III colorectal cancer. However, surgery alone results in a low 5 year disease-free survival rate [4] with half of the patients either having metastases at the time of presentation, or developing them during the course of disease [1, 5]. In this context oxaliplatin, a third generation platinum compound has remained the backbone in the treatment of colorectal cancer both in the adjuvant and in metastatic settings [6–8]. As a single agent oxaliplatin has a 5 year disease free survival rate of 10% to 20% [9–11], when combined with fluorouracil and leucovorin (FOLFOX), a progression-disease free state was observed in 58% of patients [12–15] and a 5 year disease free survival rate of 78% [7].

The side effects of oxaliplatin infusion can limit patient compliance during cancer treatment. Whilst oxaliplatin has small but notable renal, haematological and gastrointestinal toxicities [16], the emergence of cold-induced (or cold-exaggerated) neuropathic pain like symptoms during and immediately following the first treatment in 65–98% of patients predisposes this group to increasingly severe neuropathy in the subsequent cycles [17–20]. Likewise, Attal et al., have shown that the duration of cold- (and touch-) evoked pain experienced during the first three cycles were associated with the extent of chronic pain experienced one year later [21]. Studies focused on CRC have recognised acute neuropathy as a well- established risk factor for developing a persistent change in nerve function or neuropathy [17, 19, 22–27].

With recent studies demonstrating that acute neuropathy results in prolonged infusion times [in 22% of patients: 17, 25], treatment delay [in 2% of patients: 25], dose reduction [in 14.5% patients: 25, 28], treatment cessation [in 6–21% of patients: 25, 28, 29, 30, 31] and functional impairment in 43% patients [25]. It is surprising that the majority of reviews remain focused on the emergence of persistent neuropathy [e.g., 32]. Despite the large negative impact of acute neuropathy on chemotherapy [17, 25, 28–32], there is limited reporting of the factors affecting the occurrence and severity of acute neuropathy such as the starting/ the range (or minimal) doses required to evoke an acute neuropathy; numbers of patients need prolonged infusion time, dose reduction, treatment delay and treatment cessation during the acute phase (< 14 days). The current review focuses on the prevalence of acute oxaliplatin- induced neuropathy within the first treatment cycle (between start of infusion and day 14) among colorectal cancer patients treated with oxaliplatin as a monotherapy and/ or in combination with other anti-cancer drugs.

## Methods

### Data sources and search strategy

A systematic search of the literature databases of PubMed and Medline was performed using key terms ‘Colorectal Cancer’, ‘Oxaliplatin’, ‘Neurotoxicity’, ‘Oxaliplatin- Induced Acute Neuropathy’ and Oxaliplatin-Induced Chronic Neuropathy’; commenced on 13/11/ 2016. In order to minimise the loss of relevant references all identified articles were checked for other relevant publications.

### Study selection criteria

**Fig. 1:**
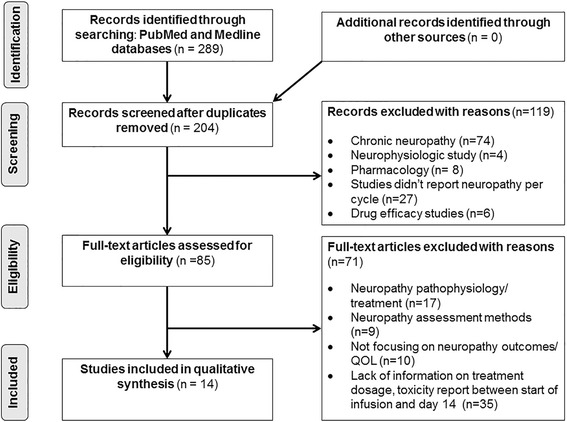
PRISMA flow diagram of included and excluded studies

Published studies that fulfilled the following criteria were included if: (1). Oxaliplatin-induced acute toxicity was assessed among cancer patients treated with oxaliplatin between the start of infusion and day 14, (2) information about oxaliplatin treatment was available (e.g. treatment schedule, starting dose, dose modification criteria, treatment compliance), (3) empirical data papers, (4) published in peer-reviewed journals and (5) written in English. Editorials, poster abstracts, reviews, preventive strategies and therapeutic studies were excluded. The inclusion and exclusion criteria were applied to the initial 289 studies published between 1992–2016 (Fig. 1). Fourteen articles met inclusion criteria were included in this review [8, 11, 14, 15, 17, 25, 29, 32–38].

### Quality assessment of studies

**Fig. 2:**
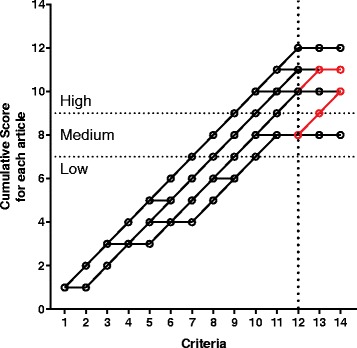
Individual plot of quality assessment for reviewed studies. Each study was initially assessed against 12 previously used criterial [39]. In the current study two additional criteria (criteria 13 and 14, as per Table 1) were included in order to assess the impact of acute neuropathy on treatment compliance in the first 14 days. The cumulative scores for successive criterion in each study are joined by a connecting line; based on the final cumulative score (criterion 14) studies were deemed to be of low (< 7), medium (7–9) and high quality (≥10). The addition two criteria revealed that only 2 of the 14 (highlighted in red) studies documented the impact of acute neuropathy in the first 14 days on treatment

Studies were quality assessed based on a scoring criterion points system adapted from a published paper [39] (see Table 1). Each item of a selected study that met the criteria received one point. If an item did not fulfil the criteria, it scored no points and the data is presented in Fig. 2 as a cumulative score. Consistent with prior criteria [39] (and as indicated by the horizontal lines in Fig. 2) studies were deemed of high quality if they scored greater than 75% of the maximum achievable score (≥10/14). Studies of adequate quality achieved a score between 50%–75% (7–9 points), and studies with a score < 7 points were classified as low quality [39]. In the current study, two additional criteria were added (see Table 1, criteria 13 and 14) to specifically assess the impact of acute neuropathy on treatment compliance in the first 14 days (Table 1).

**Table 1:** Assessment criteria for methodological quality of studies

Study scoring criteria
Measures for outcome: 1. Assessment tool used for oxaliplatin-induced toxicity is described 2. A description of oxaliplatin administration given (regimen, dose modification criteria) 3. Acute neuropathy assessment is described
Study population: 4. A description of baseline variables at least two is included (age, sex, cancer, stage) 5. Inclusion and exclusion criteria are described 6. Time of acute oxaliplatin-induced toxicity measurement and number of patients assessed are described between the initiation of infusion and day 14. 7. Information is given about study subject selection process criteria
Study design: 8. The study sample size is described 9. The data is prospectively gathered 10. The process of data collection is described
Results: 11. Acute toxicities are described 12. The cycle at which acute toxicity occurred is described 13. The number of patients who needed prolonged infusion and/ or dose modification due to acute toxicity described. 14. The number of patients who needed treatment delay and/ or cessation due to acute toxicity reported.

Criteria modified from [39]

### Statistical analysis

Descriptive statistics were employed to calculate the incidence of acute oxaliplatin-induced neuropathy.

## Results

### Study characteristics

Fourteen studies published between 1992 and 2013 were included in this review (Table 2). Study designs were prospective phase-II trials [11, 14, 15, 29, 33–35], prospective phase-III trials [36, 37], prospective follow-up studies [17, 32, 38] and one retrospective cross-sectional study [25]. Their quality scores ranged from 8–12 points (Table 2, Fig. 2). Twelve studies were ranked high quality [11, 14, 15, 17, 25, 32–37], whereas two studies were considered to be medium quality [29, 38].

**Table 2:** Characteristics and methodological quality of studies

Study author	Patients treated with oxaliplatin (n)	Study design	Oxaliplatin treatment schedule/regimen	Study quality score
Andre [14]	97	Prospective	FOLFOX3 or FOLFOX4 (85 mg/m^2^ as a 2 h infusion day1, repeated every 2 weeks)	11
Argyriou [17]	170	Prospective	FOLFOX4 (Oxaliplatin: 85 mg/m^2^ as a 2 h infusion on day1, repeated every 2 weeks)	11
Argyriou [32]	150	Prospective	FOLFOX/XELOX (OX dose) = NR	11
Davidov [38]	26	Prospective	FOLFOX (Oxaliplatin: 85 mg/m^2^ as a 2 h infusion on day1, repeated every two weeks).	8
Diaz-Rubio [11]	25	Prospective	Oxaliplatin (130 mg/m^2^ as a 2 hour infusion on day1, repeated every 3 weeks)	10
Land [36]	395/2492	Prospective	FOLFOX (Oxaliplatin: 85 mg/m^2^ IV infusion on day 1 of week 1, 3 and 5 of each 8 week cycle for three cycles)	10
Levi [15]	93	Prospective	FOLFOX (25 mg/m^2^/day infusion for 5 days, repeated every 3 weeks)	12
Pfeiffer [29]	70	Prospective	XELOX (Oxaliplatin: 130 mg/m^2^ as a 30 min infusion on day1, repeated every 3 weeks)	8
Ravaioli [33]	45	Prospective	FOLFOX (Oxaliplatin: 130 mg/m^2^ as a 2 h infusion day1, repeated every 3 weeks)	12
Rothenberg [8]	463	Prospective	Oxaliplatin (85 mg/m^2^ as a 2 h infusion on day1, repeated every 2 weeks) and FOLFOX (85 mg/m^2^ as a 2 h infusion on day1, repeated every 2 weeks)	11
Schmoll [37]	1864	Prospective	XELOX (Oxaliplatin: 130 mg/m^2^ as a 2 h infusion on day1, repeated every 3 weeks)	10
Shields [34]	48	Prospective	XELOX (Oxaliplatin: 130 mg/m^2^ as a 2 h infusion on day1, repeated every 3 week)	11
Sorbye [35]	85	Prospective	FOLFOX (Oxaliplatin:85 mg/m^2^ as a 2 h infusion day1, repeated every 2 weeks)	10
Storey [25]	188	Retrospective	XELOX (Oxaliplatin: 130 mg/m^2^ 2 h infusion on day 1, repeated every 3 weeks)	10

*FOLFOX* Folinic acid (Leucovorin); Fluorouracil; Oxaliplatin (OX), *XELOX* Capecitabine (Xeloda); Oxaliplatin (OX), *NR* Starting dose of the regimen was Not Reported

The number of patients treated with oxaliplatin in individual studies ranged from 25–2887 patients [11, 36]. The stage of cancer for those patients treated with oxaliplatin was described only in two studies [36, 37]. Oxaliplatin was administered in combination with fluorouracil (5-FU) and leucovorin/folinic acid (LV/FA) as FOLFOX regimen [8, 14, 15, 17, 29, 32, 33, 36, 38]. Moreover, combination therapy with capecitabine (Xeloda, a DNA inhibitor) as XELOX regimen was given in four studies [25, 29, 34, 37]. Oxaliplatin monotherapy was administered in one study [11], and the exact doses of each drug in the regimen (FOLFOX/XELOX) were not included in one study [32], (Table 2).

Acute oxaliplatin-induced toxicity was evaluated using several different tools. The National Cancer Institute-Common Toxicity Criteria (NCI-CTC) was the most commonly used tool for the assessment of acute neuropathy [8, 11, 14, 25, 29, 34, 37] followed by the World Health Organization (WHO) Toxicity Criteria [15, 33, 38], Neuropathy was also assessed using NCI-CTC plus nerve conduction study (NCS) [17], NCI-CTC plus Oxaliplatin Specific Neuropathy Scale (OSNS) [35], Functional Assessment of Cancer Therapy (FACT) plus OSNS [36] and NCI-CTC plus Clinical Total Neuropathy Score (TNSc) [32] (Tables 3 and 4).

**Table 3:** Dose modification criteria, acute neuropathy assessment tools and incidence of acute neuropathy

Study author	Starting dose of oxaliplatin	Dose modification criteria	Toxicity assessment tool	Acute neurotoxicity
Argyriou [17]	85 mg/m^2^	-Oxaliplatin: 30% reduction for persistent or temporary (at least 14 days) painful paresthesia, dysesthesia or functional impairment -Grade 3 persisted with 30% dose reduction, OXA omitted	-NCI-CTC v3.0 -NCS	Acute neuropathy (85.9%)
Argyriou [32]	NR	-Oxaliplatin: 30% reduction for persistent or temporary (at least 14 days) painful paresthesia, dysesthesia or functional impairment -Grade 3 persisted with 30% dose reduction, OXA omitted	-TNSc -NCI-CTC	Acute cold induced perioral dysesthesia (89.3–98.4%) and pharyngolaryngeal dysesthesia (91.7–98.3%)
Davidov [38]	85 mg/m^2^	-Oxaliplatin: 25% reduction for persistent paresthesia between cycles. Second 25% reduction if no improvement.	-WHO toxicity criteria	Acute neuropathy (58.3%), prolonged infusion (17–23.2%),
Diaz-Rubio [11]	130 mg/m^2^	−25% reduction for NCI grade 3 neutropenia, thrombocytopenia, peripheral neurotoxicity, or grade 2 renal toxicity. 50% reduction for grade 4 neutropenia, thrombocytopenia or grade 3 renal toxicity	-NCI-CTC (National Cancer Institute Common Toxicity Criteria) criteria	Laryngopharyngeal dysesthesia, and severe dyspnea 1(4%)
Land [36]	85 mg/m^2^	-Oxaliplatin: dose reduced for grade2 toxicity persisted b/n cycles or any grade 3 toxicity. Dose termination: persistent grade 3 or grade 4 toxicity	-FACT (Functional Assessment of Cancer Therapy) -OSNS (Oxaliplatin Specific Neurotoxicity Scale)	Acute neurotoxicity (68%)
Levi [15]	25 mg/m^2^	NR	-WHO haematological, skin, mucosal, & hair toxicity. Symptomatic neurological toxicity grading	Paresthesia of finger and toes in cycle Grade 1–2 (58%)
Ravaioli [33]	130 mg/m^2^	NR	-WHO toxicity criteria used	Acute neuropathy (20%)
Rothenberg [8]	85 mg/m^2^	-Dose of oxaliplatin reduced by 24% for grade 3/4 febrile neutropenia, thrombocytopenia, nausea vomiting, diarrhoea and grade4 stomatitis. Discontinue for grade 3/4 allergic reaction.	-NCI-CTC v2.0	Acute, cold-sensitive paresthesias: all grades (58%) & grades 3–4: (7%)
Schmoll [37]	130 mg/m^2^	-Oxaliplatin: 23% reduction for grade 3/4 nausea or vomiting, grade 4 stomatitis, and for paresthesias with pain or functional impairment lasting for more than 7 days, or paresthesias with pain persisting between cycles	-NCI-CTC v3.0	Grades 2–4 neuropathy on day one.
Storey [25]	130 mg/m^2^	-Oxaliplatin: Infusion prolonged for 4 or 6 h after acute, jelly legs, pseudolaryngospasm and severe laryngeal dysaesthesia.	-NCI-CTC v3.0	Acute neuropathy (94%), prolonged infusion (22%), dose reduction (14.5%), treatment delay (2%), treatment cessation (13%) & function impairment function /grade2–4 (43%)

*WHO* World Health Organization, *NCI-CTC* National Cancer Institute- Common Toxicity Criteria, *TNSc* Clinical Version of Total Neuropathy Score, *NCS* Nerve Conduction Study, *NR* Not reported

**Table 4:** Dose modification criteria, toxicity assessment tools, haematological and gastro-intestinal side effects

Study author	Starting dose of oxaliplatin	Dose modification criteria	Toxicity assessment tool	Haematological toxicity	GI toxicity
Andre [14]	85 mg/m^2^	-Oxaliplatin reduced by 25% for grade 3 thrombocytopenia or grade 4 diarrhea, and by 50% if grade 4 thrombocytopenia	-NCI-CTC	Grade 4 leukopenia, grade 3 thrombocytopenia & grade 3 anemia	Grade 4 stomatitis and grade 4 diarrhea.
Pfeiffer [29]	130 mg/m^2^	-Oxaliplatin: 25% reduction for febrile neutropenia, grade 4 thrombocytopenia or grade 3/4 GI toxicity. Additional 25% reduction if the above toxicity recurs.	-NCI-CTC v2.0	Acute grade4 neutropenia (1.4%)	Acute grade 3 stomatitis (1.4%)
Rothenberg [8]	85 mg/m^2^	-Dose of oxaliplatin reduced by 24% for grade 3/4 febrile neutropenia, thrombocytopenia, nausea vomiting, diarrhoea and grade 4 stomatitis. Discontinue for grade 3/4 allergic reaction.	-NCI-CTC v2.0	Anemia: all grades = 98 (64%) and grades 3–4 = 2(1%). Thrombocytopenia: all grades = 46 (30%) and grades 3–4 = 4 (3%). Neutropenia: all grades = 10 (7%).	- Diarrhea all grades: 70 (46%) & grades 3–4: 6 (4%). Nausea: all grades 98 (64%) & grades 3–4: 6(4%). Vomiting: all grades = 57 (37%) and grades 3–4 = 6 (4%). Stomatitis: all grade = 21 (14%).
Schmoll [37]	130 mg/m^2^	-Oxaliplatin: 23% reduction for grade 3/4 nausea or vomiting, grade 4 stomatitis, and for paresthesias with pain or functional impairment lasting for more than 7 days, or paresthesias with pain persisting between cycles	-NCI-CTC v3.0	Grade 3/4 neutropenia (4-20%)	acute grade 3/4 diarrhea on day1 (19%).
Shields [34]	130 mg/m^2^	Oxaliplatin: 25% reduction for grade 3 thrombocytopenia, grade4 neutropenia mucositis & diarrhea, grade 3/4 emesis and paresthesia persisting b/n cycles. 40% for grade 4 thrombocytopenia and 50% for paresthesia impairing function	-NCI-CTC v2.0	NR	Diarrhea 1 (7.7%)
Sorbye [35]	85 mg/m^2^	-Oxaliplatin: 25% reduction for persistent paresthesia b/n cycles. Second 25% reduction if no improvement	- NCI-CTC v2.0 -Oxaliplatin specific neurotoxicity scale	Acute grade 4 leukopenia 1 (1.2%) Acute allergic reaction 1 (1.2%)	Acute stomatitis 1 (1.2%)

*NCI-CTC* National Cancer Institute- Common Toxicity Criteria, *NCS* Nerve Conduction Study, *NR* Not reported

### Acute oxaliplatin-induced neuropathy

**Fig. 3:**
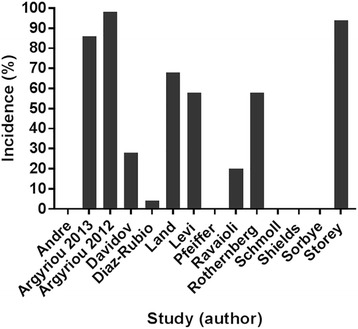
Reported incidences of acute neuropathy in the first cycle (≤14 days). Nine of 14 studies reported acute neuropathy symptoms in 4–98% of patients. In other studies, the incidence of neuropathy was not clearly identified. One other study, neuropathy was reported as grade 2–4 (percentage value was not reported) [37]

The incidence of acute neuropathy varied across studies from a low of 4% to a high of 98%. This is likely to be due to differences in the starting doses of oxaliplatin, differing drug combinations and dosing intervals (Table 3 and Fig. 3). However, no study examined the range (or minimal) dose required to evoke an acute neuropathy; rather they relied on a fixed dose regimen. Notably, even when the starting dose was at its lowest (25 mg/m^2^), 58% of patients developed grades 1–2 acute paraesthesia in the fingers and toes [15]. Moderate to severe acute oxaliplatin induced neuropathy symptoms (grades 2–4) were very common in patients who were given large starting dose of oxaliplatin (> 85 mg/m^2^) [25, 37], occurring within 24 h of treatment initiation. Consequently, dose modification criteria for the reduction and treatment of toxicity after starting the therapy were incorporated in the majority of studies [8, 11, 14, 17, 25, 29, 32, 34–38], but were not explained in two studies [15, 33] (Table 3). Only one study reported the number of patients who received reduced doses, or needed treatment delay or dropped out due to acute neuropathy [25] (Table 3).

### Haematological and gastro-intestinal side effects

Oxalipaltin treatment caused haematological toxicity in 1.4–81% patients [14, 29, 35] and gastrointestinal toxicity in 1.2–67% patients [8, 14, 29, 35, 37] respectively (Table 4). Patients treated with XELOX developed grade 3/4 diarrhoea on day one [37]. In a prospective, multicenter phase II study that evaluated the efficacy and safety of oxaliplatin combined with the Nordic bolus schedule of fluorouracil (5-FU) and folinic acid (FA) as first-line treatment in metastatic CRC, acute allergic reaction occurred in 1% (1/85), grade 4 leukopenia in 1% (1/85) and stomatitis in 1% (1/85) patients respectively [35]. Likewise, in another study, patients developed grade 1 leukopenia, grade 1 thrombocytopenia and grade1 anemia, grade 1 stomatitis and grade1 diarrhoea respectively [17]. In addition, in a prospective phase II study, grade 4 neutropenia and graded 3 stomatitis occurred in 1.4% (1/70) and 1.4% (1/70) patients respectively [29] (Table 4).

## Discussion

Acute OXIPN occurs in the majority of patients treated with oxaliplatin and is considered to be a transient event that resolves in the first cycle [17]. However, recent studies have shown that a large number of patients continue to experience acute neuropathic pain like symptoms that tend to be more severe in cycle 2 and follow the same pattern in the remaining cycles [8, 32, 40]. Moreover, we have emphasised the impact of acute neuropathy on dose regimens, namely that the emergence of oxaliplatin-induced acute neuropathy caused prolongation of infusion times in 12–22% patients [17, 25, 32], and/ or dose reduction in 15–43% patients [25, 28], and/or treatment cessation in 6–21.4% patients [25, 28, 30, 31] with functional impairment in 43% of patients [25].

This review focused on factors that affect the occurrence and severity of acute neuropathy such as treatment regimen, dose reduction criteria, starting/the range (minimal) doses of oxaliplatin required to evoke an acute neuropathy, patient related and clinical risk factors. We also assessed the number of patients where prolonged infusion time, dose reduction, treatment delay and treatment cessation were implemented, and neuropathy assessment tool. Even though the majority of studies described the type of drug regimen and starting dose of each drug in the treatment regimens, no single study reported the actual dose (i.e. how much of the starting dose was received by the patient) that caused acute toxicity during the acute phase. Notably, the lowest dosing regimen (25 mg/m^2^/day for five days) was associated with a low incidence (4%) of acute neuropathy [15] compared to high dose regimens (86%, 85–130 mg/m^2^ on day one) [17]. Underreporting of such important parameters can result in premature treatment adjustment and negatively impacts on the clinical decision making process [17].

It is well established that the risk of developing chronic OXIPN is correlated with the treatment schedule, duration of infusion, starting dose of oxaliplatin, severity of acute toxicity, cumulative dose, patient and clinical factors [41]. While this may be repeatedly stated in many reviews [39, 42], only 9/14 studies reviewed here reported the incidence of acute neuropathy in the first cycle. Furthermore, even though most studies applied dose reduction criteria in order to limit the degree of subsequent toxicity, the number of patients who received reduced dose, treatment delay and treatment cessation due to acute neuropathy was reported in one study only [25]. The lack of a systematic, detailed approach to presentation of the number of patients who received dose reduction (and when) and dropout rates within the first cycle, means that the impact of such parameters on subsequent treatment cannot be informed by comprehensive data sets, making dose modification difficult in order to limit the development of acute and chronic neuropathies [43].

In this review, no study reported the number of patients whose symptoms resolved and those who had persistent neuropathy in the second cycle among who developed acute neuropathy in the first 14 days of chemotherapy. This will hamper preventive actions and treatment optimization at the early stage of treatment [43]. Moreover, studies differed in starting dose (duration of infusion, amount/ total dose), type of combination of drugs in each regimen, study design, type of cancer patients (chemonaïve/ previously untreated), neuropathy assessment tool, time of assessment of toxicity after treatment initiation and result presentation (acute versus chronic, time of occurrence of toxicity, degree of severity of symptoms, and measures taken). These heterogeneities across studies could hinder the early prediction of acute neuropathy, treatment adjustment and prevention of the ongoing development of chronic neuropathy [17].

This review also observed that severe acute neuropathy and other toxicities were common in patients treated with a large single dose of oxaliplatin (> 85 mg/m^2^) and/or combined drugs in the treatment regimens. As the incidence of neuropathy observed when oxaliplatin was given alone, or in combination, were overlapping, it is difficult to ascertain whether the degree of neuropathy was due to synergetic drug effects, and this requires more studies that systematically document the emergence of neuropathy in cycle 1 (≤ 14 days). In a phase III trial that compared XELOX with bolus FULV as adjuvant therapy for stage III CRC with a starting dose of oxaliplatin 130 mg/m^2^, capecitabine 1000 mg/m^2^, leucovorin 500 mg/m^2^ and fluorouracil 500 mg/m^2^, patients developed acute grade2–4 neurotoxicity and 19% (178 /938) experienced grade 3/4 diarrhoea [37]. In addition, in a retrospective cross-sectional study that compared the incidence of acute neuropathy between XELOX and FOLFOX with a starting dose of oxaliplatin 130 mg/m^2^ and capecitabine 1000 mg/m^2^, the overall incidence of acute neuropathy in oxaliplatin treated group was 94% and 43% of patients developed grade 2–4 neuropathy that impaired daily function [25]. In these studies, severe neuropathy and gastrointestinal adverse effects that occurred could be attributed to large doses and the combined effects of drugs in the treatment regimens.

There was also considerable variation of the assessment tool used to identify oxaliplatin- induced acute toxicity across studies, although NCI-CTC was the commonest tool employed. Therefore, a lack of standardized assessment tool will underestimate the prevalence of acute toxicity and makes comparison among studies difficult [44]. Moreover, comparing the prevalence and severity of neuropathy even using NCI-CTC is still difficult as there is a the potential for interobserver disagreement [45]. Furthermore, there is no consensus whether subjective or objective assessment methods are important to determine the severity of both acute and chronic neuropathies [39].

Given the lack of well-proven neuroprotective agents or treatment options for acute oxaliplatin induced neuropathy, it is paramount to identify risk factors [23, 46]. Even if a potential neuro-protective treatment can be identified, the emergence of acute neuropathies during the first treatment cycle highlights the need for a pre-emptive intervention prior to the first dose of oxaliplatin. Whether these acute hypersensitivities, presumed to be the result of neuronal sensitisation, are mechanistically distinct from the emergence of persistent neuropathy following repeated doses of oxaliplatin cannot be resolved with the available data. Likewise, none of the studies reviewed here discussed risk factors. Rather, some authors excluded patients with risk factors such as pre-existing peripheral neuropathy, diabetes mellitus, and alcohol abuse to avoid interference with their clinical assessment [17, 32, 36]. Oxaliplatin-induced peripheral neuropathy has a major negative impact on the quality of life of CRC patients. Therefore, it will be of great value to understand the patient and clinical related risk factors such as intensity of acute symptoms, duration of cold-evoked pain in the past, body surface area < 2.0 m^2^, winter-period, pre-existing neuropathy, previous or co-administered toxic chemotherapeutic drugs and diabetes mellitus [42].

Evidence shows that, oxalate, a metabolite of oxaliplatin alters the functional properties of voltage gated sodium channels in DRG neurons that leads to change in channel function causing hyperexcitability of sensory neurons [47–50]. Moreover, indirect interactions with voltage-gated sodium channels, through chelation of intracellular calcium can cause membrane hyperexcitability [26]. Acute hyperexcitability is a strong mediator or predictor of oxaliplatin induced chronic peripheral nerve damage [17]. Therefore, treating physicians may be advised to adjust the doses based on the severity of neuropathy- like symptoms and /or patients’ conditions, and closely monitor patients using standardized neuropathy assessment tools to minimise the severity of acute neuropathy, improve treatment compliance and to prevent the ongoing development of chronic neuropathy. Furthermore, nerve excitability studies may provide additional objective assessment for acute neurotoxicity following the initiation of infusion and ongoing development of chronic/cumulative neurotoxicity [46, 51]. However, while these techniques have been applied in research studies this has not translated to routine clinical practice.

## Conclusion

In the current review, studies varied regarding starting dose of oxaliplatin, treatment regimens, study design, acute neuropathy assessment tool and result presentation (acute versus chronic and measures taken). Despite the heterogeneity of studies, a large number of patients developed acute neuropathy, and moderate to severe toxicities were relatively common in patients received single large dose of oxaliplatin (> 85 mg/m^2^) and combined drugs in the treatment regimens.

In addition, the majority of studies did not report the factors that affect the occurrence and severity of acute neuropathy (< 14 days) such as the minimal dose required to evoke an acute neuropathy, patient related and clinical risk factors. Likewise, there was no systematic reporting of the number of patients subjected to prolonged infusion, dose reduction, treatment delay and treatment cessation during the acute phase.

Recent studies reveal that a large number of patients continue experiencing acute neuropathic symptoms until cycle 2 [8, 32, 40]. The persistence of these acute neuropathic symptoms results in subsequent prolongation of infusion time, or dose reduction and/ or treatment cessation nearly in quarter of patients during the acute phase. To develop better preventive and therapeutic guideline for acute/chronic neuropathy, a prospective study should be conducted in a large cohort of patients in relation to drug regimen, starting/ the ranges of oxaliplatin dose producing acute neuropathy, treatment compliance, patient and clinical risk factors using a standardised neuropathy assessment tool. Moreover, oncologists should monitor patients routinely during clinical assessment and use a standardised neuropathy assessment tool in order to detect acute neuropathy early, improve treatment compliance and to prevent/ameliorate the development of persistent neuropathy. Furthermore, nerve excitability tests need to be considered for patient monitoring as it may provide additional objective information for the assessment of acute hyperexcitability following the administration of oxaliplatin.

## Abbreviations

FACT: Functional Assessment of Cancer Therapy
FOLFOX: Folinic Acid (Leucovorin) (FOL), Fluorouracil (F), Oxaliplatin (Ox)
NCI-CTC: National Cancer Institute- Common Toxicity Criteria
NCS: Nerve Conduction Study
OSNS: Oxaliplatin Specific Neurotoxicity Scale
TNSc: Clinical Version of Total Neuropathy Score
XELOX: Capecitabine (Xeloda) and Oxaliplatin (OX)
